# Favorite Parts of a Single Leaf for Giant Flying Squirrels to Eat in Three Species of Food Trees

**DOI:** 10.3390/biology12101352

**Published:** 2023-10-22

**Authors:** Mutsumi Ito, Noriko Tamura, Fumio Hayashi

**Affiliations:** 1 Department of Biology, Tokyo Metropolitan University, 1-1 Minamiosawa, Hachioji, Tokyo 192-0397, Japan; fhayashi@tmu.ac.jp; 2 Faculty of Science and Engineering, Chuo University, 1-13-27 Kasuga, Bunkyo, Tokyo 112-8551, Japan; 3 Tama Forest Science Garden, Forestry and Forest Product Research Institute, 1833-81 Todori, Hachioji, Tokyo 193-0843, Japan; haya@ffpri.affrc.go.jp

**Keywords:** feeding behavior, folivory, food selection, glucose content, total phenolic content

## Abstract

**Simple Summary:**

Herbivorous mammals are considered to eat plants with high nutrition but to avoid those with harmful chemical components. Leaf chemical components differ between plant species, conspecific individuals, leaves of the same individual, and the parts of a single leaf; therefore, food selection by herbivores (particularly browsers) occurs in these hierarchical structures. However, the effects of plant chemicals on food selectivity are not well known in the tree-leaf-eating mammals such as arboreal primates, rodents, and marsupials, as compared with other mammals eating herbaceous plants, seeds, nuts, and fruits. Moreover, the effects of the microscale chemical distribution within the single leaf on their partial feeding have been little examined. The Japanese giant flying squirrel (*Petaurista leucogenys*) is an arboreal herbivorous mammal that feeds on mostly tree leaves. In our study site, the three tree species, evergreen *Phonitia serratifolia*, deciduous *Quercus acutissima*, and evergreen *Q. sessilifolia*, were used by this flying squirrel as main food. In comparisons of feeding preference and chemical (phenolic, glucose, and water) contents of leaves among these tree species, leaf glucose concentration was an important factor determining which species of tree leaves they eat. In comparisons between favorite parts of consumption and chemical contents within the single leaf, the central consumption was frequent when the water contents were higher at the leaf center than the margin. Phenolic contents in the central and marginal parts of the single leaf differed among the three tree species, but clear tendency to avoid eating the part with high phenolic contents was not detected.

**Abstract:**

To examine the effect of leaf chemical composition on selective herbivory by the Japanese giant flying squirrels (*Petaurista leucogenys*), we measured and compared the total phenolic, glucose, and water contents of leaves among their main food tree species, deciduous *Quercus acutissima*, and evergreen *Q. sessilifolia* and *Phonitia serratifolia*. Leaves of these three tree species were available in the warm season (April to October), but the flying squirrels mostly preferred the leaves of *Q. acutissima,* having higher glucose and water contents than those of the other two tree species. In the cold season (November to the next March), the two evergreen tree species were available, and the flying squirrels used both leaves without any apparent influence of the chemical compositions. On the other hand, the favorite parts of a single leaf differed among the three tree species. Flying squirrels dropped the individual leaves after partial consumption. Their feeding marks on the dropped leaves were distinguished into four types: apical, basal, central, and marginal parts of consumption. The basal parts of consumption were most frequent in *Q. acutissima* leaves in which more water was contained at the basal part, and the central part consumption followed, which may be related to a lower phenolic content and more glucose and water at the leaf center than its margin. In contrast, the apically consumed leaves dominated in *Q. sessilifolia,* with relatively homogeneous leaf chemical distribution except for more water at the center. In *P. serratifolia*, leaves consumed at the center were frequent, but those with marginal consumption were also observed, which may be related to its specific chemical distribution with less phenolics and more glucose at the leaf margin. Thus, the chemical distributions within the single leaf differ among tree species, and the flying squirrel’s selectivity of the tree species and the part of each leaf depends partly on the relative compositions of preferable glucose and water and unpreferable phenolics.

## 1. Introduction

Plants contain a variety of chemicals that affect the food choice of herbivorous mammals to obtain sufficient nutrition and to avoid toxicity. Sugars, proteins, and minerals are preferred by herbivores as the main nutritional components obtained from plants, but low-energy, indigestible fibers (e.g., cellulose, hemicellulose, and lignin), toxic secondary metabolites (phenols, alkaloids, etc.), and digestion inhibitors (e.g., condensed tannins) are avoided [1,2,3,4,5]. Among plant body parts, leaves are abundant food resources during plant growing seasons and are always in evergreen plants, compared to temporally available seeds, flowers, and fruits.

Some groups of herbivorous mammals are specialized tree leaf foragers. Tree leaves are primarily composed of indigestive fibers, making them a low-energy resource [1,2], and some arboreal mammals, such as primates and rodents, can use such fibers and other plant materials by converting them into absorbable nutrients by specialized intestinal flora [6,7]. Tree leaves are also rich in the defensive secondary metabolites [8]. The generalist herbivores often consume small amounts of leaves of various tree species. Such foraging may minimize the risk from leaves with unknown toxins and contribute to toxicity identification through continuous sampling [9,10,11]. In contrast, specialist herbivores feed on only a limited range of tree species and have a tolerance for their harmful secondary metabolites [9,10].

The chemical components of tree leaves differ between tree species, conspecific individuals, and leaves of the same individual; therefore, food selection by herbivores occurs in these hierarchical structures [4]. Although rarely studied, selective feeding of tree leaves occurs on a much finer scale. An example is the partial consumption of a single leaf by the Japanese giant flying squirrel *Petaurista leucogenys*, despite all parts being likely to be edible [12,13]. This flying squirrel is arboreal, nocturnally active, a generalist herbivore (up to 1.3 kg body weight), and feeds primarily on tree leaves throughout the year [14]. They eat only the apical half, basal half, or central part of the leaf. Consumption of the leaf center is acquired when the flying squirrel eats the corner of the folded leaf at least two times, first along the primary vein and next in the opposite direction [4,12]. This complicated feeding manner making a hole in the leaf center varies in frequency among local populations, suggesting that it needs to be learned and transmitted among individuals [12]. Frequencies of the central consumption also differ between two sympatric species of *Quercus* trees [13]. The comparisons of their microscale distributions of chemicals in a single leaf predict that phenolic concentrations (probably related to bitterness) and water contents (probably related to hardness) affect which parts the flying squirrels eat, and glucose content (probably related to sweetness) is an important contributor to which species of tree leaves they select as food [13].

Although not leaves, partial consumption is known when some rodents consume each acorn of a *Quercus* tree [15,16]. Gray squirrels, *Sciurus carolinensis*, tend to consume only the basal part of the acorn that contains less phenolics than the apical portion where the embryo is located [15]. Wood mice and chipmunks also frequently consume the basal half of acorns, although the contribution of chemical compositions (crude protein, fat, tannin, and starch) to selectivity is still unclear [16]. If not consumed intensively, the partially consumed acorns can germinate [15,16].

In this study, first, the leaf partial consumption of the evergreen tree *Phonitia serratifolia* by Japanese giant flying squirrels is described by comparing it with two previously studied *Quercus* trees, deciduous *Q. acutissima* and evergreen *Q. sessilifolia*, that coexist and are used as the main food resources by the flying squirrels in the same study site [12,13]. Next, to extend our previous prediction that microscale distributions of phenolics and water within individual leaves affect which parts the flying squirrels eat, whereas leaf glucose concentration is an important factor affecting which species of tree leaves they eat [13], within-leaf distributions of phenolics, glucose, and water are compared among these three tree species.

## 2. Materials and Methods

### 2.1. Leaf Debris Collection

**Figure 1:**
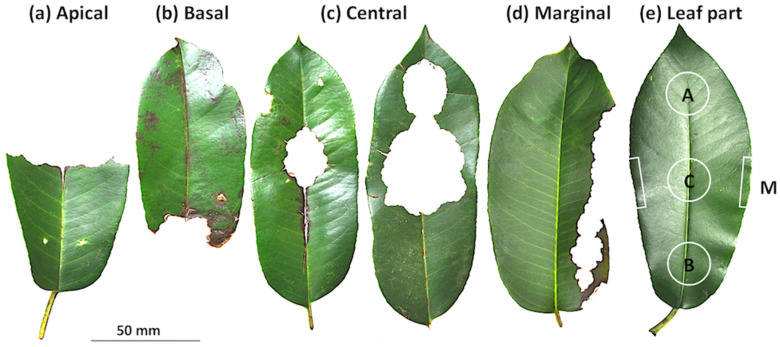
Leaf debris of the tree *Phonitia serratifolia* consumed by the Japanese giant flying squirrels *Petaurista leucogenys*. Four types, (**a**) apical, (**b**) basal, (**c**) central, and (**d**) marginal feeding patterns, are distinguished. Four parts of a single leaf were cut for chemical analysis, as shown in (**e**) (A: apical disk; B: basal disk; C: central disk; M: marginal parts).

The study site encompasses 50 ha in an isolated section of the Tama Forest Science Garden at Todori, Hachioji, Tokyo, central Japan (35°38′50.74″ N, 139°16′38.15″ E). This flying squirrel was the only mammal species eating tree leaves in the study site [17]. The vegetation consists of mostly temperate broadleaf trees and partly planted coniferous trees [18]. From April 2013 to November 2015, we conducted one to five morning censuses per month along a fixed census route (2 km long, 5 m wide, a total of 87 censuses). The morning census was conducted usually from 9 a.m. along this route, walking slowly for about 1.5 h. Along the census route, we collected and counted all *Q. acutissima* and *Q. sessilifolia* leaf debris eaten by the flying squirrels. Before eating the leaf, the flying squirrels use their teeth to cut off the branch or petiole, leaving a diagonal cut there, and they usually fold leaves before eating, leaving folded marks on the eaten leaves [4,12,13]. From these features, we can judge that those leaves are not from insects but from flying squirrels. Along the way of this study, we noticed that leaves of another tree species, *Phonitia serratifolia* (two trees), standing outside, but near, the fixed census route, were used as one of the main foods. After that, collection of leaf debris of these two trees were conducted one to three times per month from December 2015 to April 2018, except for May, June, and August in 2017 (a total of 48 censuses). Collected leaf debris was classified into four types (apical, basal, central, and marginal) of feeding patterns (Figure 1a–d).

### 2.2. Leaf Chemical Analysis between Summer and Winter

Fresh leaves were collected from the branches of two trees of *P. serratifolia* (6 January 2016 and 19 July 2017), three trees of deciduous *Q. acutissima* (19 June 2016), and three trees of *Q. sessilifolia* (20 June 2016 and 3 February 2017) at 10:30 to 11:00 on clear days. Young leaves were avoided because of their different chemical compositions from mature leaves [13]. Leaves in June to July were used for summer data, and those in January to February for winter data. Collected fresh leaves were placed in an ice box for 1 h during transport and then stored at −30 °C in a laboratory freezer.

Circular disks (17 or 20 mm in diameter) were cut with a cork borer from the central part of the five fully expanded, uninjured leaves and used to determine the total phenolic concentration of each leaf disk. The Folin–Ciocalteau method was used to measure the total concentration of phenolic compounds [19]. The circular disks were dried at 60 °C overnight and then weighed to the nearest 0.1 mg. Each dried disk was powdered in a 2.0 mL tube using a pestle and mixed with 1 mL 70% aqueous acetone. The homogenate was sonicated for 10 min and centrifuged at 2500× *g* for 10 min. The supernatant was stored in a 15 mL tube. This procedure was repeated three times, and the entire 3 mL aqueous acetone extract was fully evaporated under low pressure at 40 °C. The residue was dissolved in 1 mL distilled water. After being diluted 60 times, 0.3 mL of the solution was mixed with 0.3 mL of 2N Folin reagent (Folin–Ciocalteau’s phenol reagent) in a 2.0 mL tube, allowed to stand for 5 min, and then mixed with 0.6 mL 20% Na_2_CO_3_. After a 10 min incubation at room temperature, the mixture was centrifuged at 1500× *g* for 8 min, and the absorbance at 730 nm was measured using a spectrophotometer (DU 640; Beckman Instruments, Fullerton, CA, USA). The standard curve was prepared with six concentrations of 0 to 50 mg/L gallic acid, and the phenolic contents were expressed as gallic acid equivalent (mg/g dry weight) [12].

To estimate leaf glucose and water contents, the same-sized circular disks were cut from the central part of additional five leaves immediately after being defrosted. The fresh weight of each disk was measured to the nearest 0.1 mg after excess water was wiped off with a paper towel. The disk was placed in 2 mL tubes containing 0.5 mL distilled water. After boiling at 90 °C for 20 min, the leaf sections were pulverized using a homogenizer. The tip of the homogenizer was washed with 0.5 mL distilled water, which was added to the same tube. The mixture was vortexed and then centrifuged at 1500× *g* for 10 min, and the supernatant was transferred into a new 2 mL tube. The glucose concentration of 10 μL of the supernatant was then measured with a glucose measuring instrument (Glutest Every, Sanwa Kagaku Kenkyusho Co., Ltd., Nagoya, Japan). The precipitate in the tube was dried at 80 °C for 1 day to measure the dry weight. Water contents were thus calculated as (fresh weight − dry weight)/dry weight. Since the glucose concentration in the glucose measuring instrument used in this study was overestimated depending on the phenolic concentration in the sample, a calibration curve was formulated using a glucose-free solution including known total phenolic concentration (*n* = 8, *r* = 0.97, *p* < 0.001). Thus, the overestimated glucose concentration was corrected by the equation *z* = *y* − (1.1*x* − 4.9); where *z* is the corrected glucose concentration, *y* is glucose concentration before correction, and *x* is the total phenolic concentration (mg/g dry weight in all variables). If a negative value was taken after correction, the glucose concentration was treated as zero. Glucose contents reported in our previous papers [4,12,13] were overestimated and corrected in this paper.

### 2.3. Microscale Distribution of Leaf Chemicals

To determine the distribution of the total phenolic, glucose, and water concentrations in the single leaf, circular disks (17 or 20 mm in diameter) were cut with a cork borer from the apical, central, and basal parts of the leaf, but only from apical and basal parts for some of *Q. acutissima* and all of *Q. sessilifolia* (Figure 1e). The marginal disks of the left and right sides of leaves were also cut (Figure 1e). Leaves were collected from two trees of *P. serratifolia*, three trees of *Q. acutissima*, and three trees of *Q. sessilifolia* from May in 2013 to July in 2017, and their chemical contents were analyzed by the same methods as in chemical comparisons between summer and winter.

### 2.4. Statistics

We ensured that the assumptions of the statistical tests were met by checking the normality of the data, equal variances across groups, and independence of the observations [20]. Interspecific differences in the frequencies of the four types of leaf debris found in the field censuses were tested using the multiple chi-square (*χ*^2^) tests between three tree species after Bonferroni correction for multiple comparisons. Chemical concentrations were compared between seasons, tree species, and leaf parts using the data of all leaves examined (*n*). Differences in the mean chemical concentrations between conspecific summer and winter leaves were tested by Student’s or Welch’s *t*-tests according to *F*-tests of data variance. The Kruskal–Wallis test and the following Steel–Dwass multiple comparison tests were used for summer chemical concentrations among three tree species, and the Wilcoxon rank sum test was used for winter data between two evergreen tree species. Microscale distributions of chemicals were compared between apical and basal parts of individual leaves using the paired *t*-tests, because data at the central part were not all available. The chemical comparisons between central and marginal parts of leaves were also tested by the paired *t*-tests. These tests were performed using the base package of R 4.1.2 [21].

## 3. Results

### 3.1. Seasonal Changes in Leaf Debris

**Figure 2:**
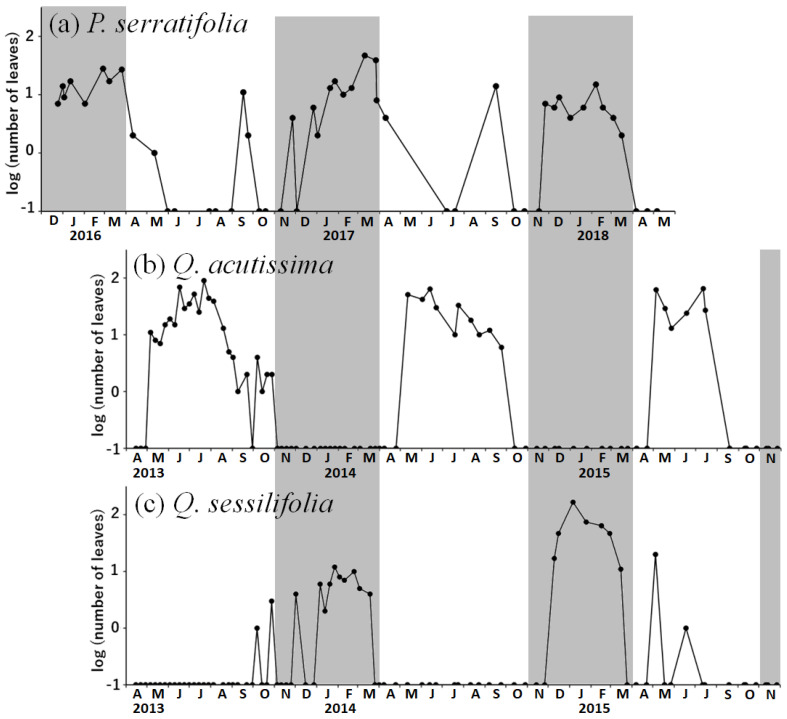
The log_10_-transformed number of leaf debris of (**a**) *Phonitia serratifolia*, (**b**) *Quercus acutissima*, and (**c**) *Q. sessilifolia* found in each census during the study period. When no leaf debris was observed, it is treated as 0.1 (−1 on log_10_ axis). The period when deciduous *Q. acutissima* has no leaves (November to the next March) is shaded.

We collected a total of 496 leaf debris of evergreen *P. serratifolia* in 48 census days (10.3 per day), 1001 debris of deciduous *Q. acutissima* in 57 census days excluding its defoliation period from November to the next March (17.6 per day), and 520 debris of evergreen *Q. sessilifolia* in 87 census days (6.0 per day). Leaf debris of *P. serratifolia* were found throughout the year, although the frequency decreased greatly in summer (May to August) (Figure 2a). In *Quercus* trees, leaves of *Q. acutissima* were available from late April to October and were eaten by the flying squirrel during this period (Figure 2b), but leaves of *Q. sessilifolia* were not used as food during the time that leaves of *Q. acutissima* were available (Figure 2c).

### 3.2. Feeding Pattern

**Figure 3:**
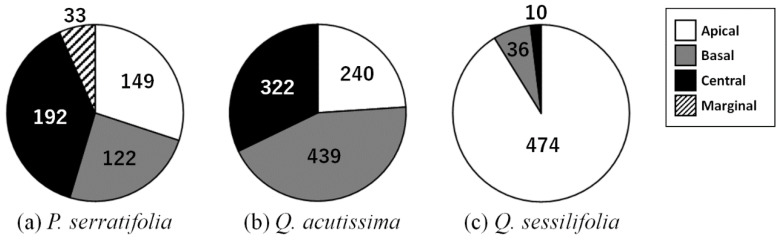
Frequency of four (apical, basal, central, and marginal) types of leaf debris in (**a**) *Phonitia serratifolia*, (**b**) *Quercus acutissima*, and (**c**) *Q. sessilifolia*. Numerals shown in the figure are the total number of each leaf debris type during the study period.

Frequencies of the four types of leaf debris differed among the three tree species (Figure 3, *χ*^2^ = 811.9, df = 6, *p* < 0.001) and also between any species combinations by multiple comparisons between tree species (*p* < 0.05 after Bonferroni correction). In *P. serratifolia*, all apically, basally, centrally, and marginally consumed leaves were found, but were most common in the central type (Figure 1c and Figure 3a). In *Q. acutissima*, apical, basal, and central types of leaf debris were found and the basal one was the most frequent (Figure 3b). In contrast, apically consumed leaf debris dominated (91.1% of the total) in *Q. sessilifolia* (Figure 3c).

### 3.3. Leaf Chemicals between Summer and Winter

In evergreen *P. serratifolia*, the mean total phenolic content was 18.38 mg/g dry weight (SD = 3.18, *n* = 10) in summer and 12.34 mg/g dry weight (SD = 3.69, *n* = 10) in winter. The mean glucose content was 13.02 mg/g dry weight (SD = 4.68, *n* = 10) in summer and 34.57 mg/g dry weight (SD = 9.31, *n* = 10) in winter. The mean water content was 1049.50 mg/g dry weight (SD = 199.76, *n* = 10) in summer and 998.99 mg/g dry weight (SD = 130.83, *n* = 10) in winter. Thus, leaves of *P. serratifolia* contained less phenolics and more glucose in winter than in summer (*t* = 3.91, df = 18, *p* < 0.01 and *t* = −6.54, df = 18, *p* < 0.0001, respectively), but similar water contents seasonally (*t* = 0.67, df = 18, *p* = 0.51).

In evergreen *Q. sessilifolia*, the mean total phenolic content was 33.70 mg/g dry weight (SD = 10.32, *n* = 15) in summer and 31.27 mg/g dry weight (SD = 4.85, *n* = 15) in winter. The mean glucose content was 0.10 mg/g dry weight (SD = 0.17, *n* = 15) in summer and 0.24 mg/g dry weight (SD = 0.42, *n* = 15) in winter. The mean water content was 1096.32 mg/g dry weight (SD = 171.84, *n* = 15) in summer and 891.01 mg/g dry weight (SD = 74.79, *n* = 15) in winter. Thus, in *Q. sessilifolia*, total phenolic and glucose contents did not differ between summer and winter leaves (*t* = 0.82, df = 20, *p* = 0.41 and *t* = −0.54, df = 19, *p* = 0.60, respectively), but the water content was slightly higher in summer (*t* = 4.24, df = 19, *p* < 0.001).

**Figure 4:**
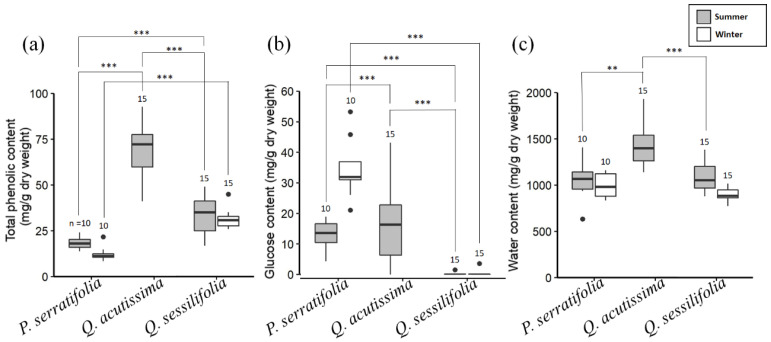
Box plots of total phenolic (**a**), glucose (**b**), and water (**c**) contents at the central part of leaves of *Phonitia serratifolia*, *Quercus acutissima*, and *Q. sessilifolia* in summer (shaded bars) and winter (white bars); *n*: total number of leaves examined; ** *p* < 0.01, *** *p* < 0.001 in Steel–Dwass multiple comparison tests for summer data and in Wilcoxon rank sum tests for winter data. Note that leaves of deciduous *Q. acutissima* are unavailable in winter.

Among three tree species in summer, total phenolic contents were highest in deciduous *Q. acutissima* and lowest in *P. serratifolia* (Figure 4a), and glucose contents were highest in *Q. acutissima* and lowest in *Q. sessilifolia* (Figure 4b). Water contents were higher in *Q. acutissima* than in the other two species (Figure 4c). Between evergreen two tree species in winter, leaves of *P. serratifolia* contained lower phenolic and higher glucose contents than those of *Q. sessilifolia* (Figure 4a,b). Leaf water contents were similar between these two species (Figure 4c).

### 3.4. Microscale Distributions of Leaf Chemicals

**Figure 5:**
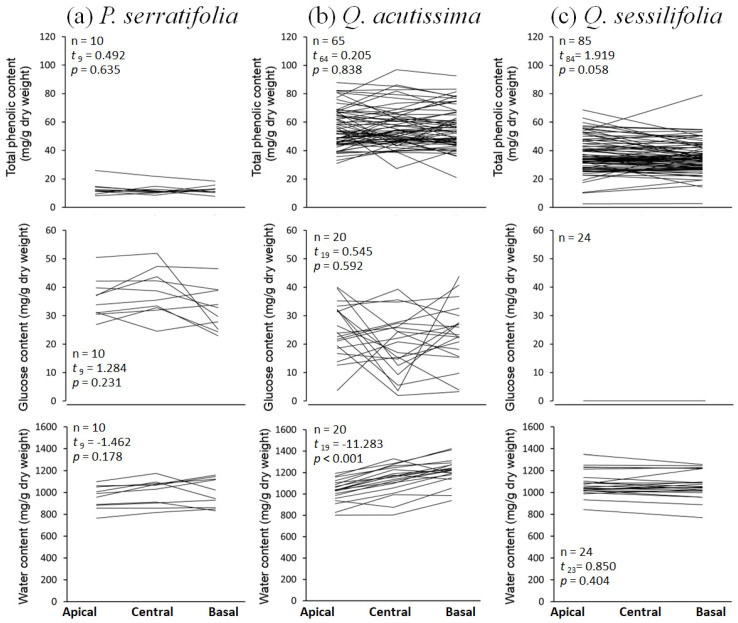
Total phenolic, glucose, and water contents at apical, central, and basal parts of a single leaf (see Figure 1e) of (**a**) *Phonitia serratifolia*, (**b**) *Quercus acutissima*, and (**c**) *Q. sessilifolia*; *n*: total number of leaves examined. The results of paired *t*-tests comparing chemical contents between the apical and basal parts are shown (data at the central part are not all available). The mean concentration at the apical part is higher if *t* > 0, and lower if *t* < 0.

**Figure 6:**
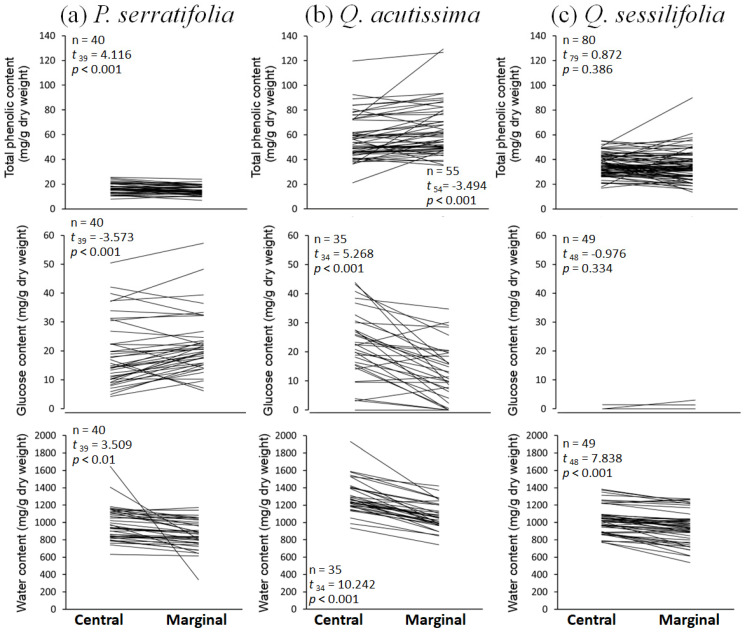
Total phenolic, glucose, and water contents at the central and marginal parts of a single leaf (see Figure 1e) of (**a**) *Phonitia serratifolia*, (**b**) *Quercus acutissima*, and (**c**) *Q. sessilifolia*; *n*: total number of leaves examined. The results of paired *t*-tests comparing chemical contents between the central and marginal parts are shown. The mean concentration at the central part is higher if *t* > 0, and lower if *t* < 0.

Phenolic, glucose, and water contents did not differ between the apical and basal parts of the single leaf in all three tree species, except for the water content in *Q. acutissima* leaves in which the basal part contains more water (Figure 5). In contrast, chemical contents differed between the leaf center and margin. In *P. serratifolia*, phenolic and water contents were lower, but the glucose content was higher at the leaf margin (Figure 6a). In *Q. acutissima*, the phenolic content was higher, but glucose and water contents were lower at the leaf margin (Figure 6b). Leaves of *Q. sessilifolia* did not show the different phenolic and glucose contents between the center and margin, but there was lower water content at the margin (Figure 6c). Thus, the phenolics and glucose showed species-specific trends between the leaf center and margin, whereas the water tended to decrease at the leaf margin in all tree species (Figure 6), or occasionally from basally to apically as observed in *Q. acutissima* (Figure 5).

## 4. Discussion

### 4.1. Preferred Tree Species and Chemical Composition

A great amount of leaf debris of *P. serratifolia*, *Q. acutissima*, and *Q. sessilifolia* suggests that leaves of these three tree species are the main food resources for the flying squirrels in the study site. It is usually difficult to estimate the density of nocturnally active flying squirrels. Females of this flying squirrel have nonoverlapping home ranges (0.4–2.68 ha in size) and males have overlapping ranges (0.78–5.16 ha in size) [14]. Before starting our study, 2.2–3.9 flying squirrels/ha could be seen at night in this study site [22].

Leaves of the three tree species were selectively used as food (Figure 2). During April to October, when all three species were available, leaves of *Q. acutissima* were commonly used and those of *P. serratifolia* were occasionally used, but those of *Q. sessilifolia* were rarely used. During November to the next March, when leaves of deciduous *Q. acutissima* were unavailable, the squirrels used both *P. serratifolia* (10.3 leaf debris/census) and *Q. sessilifolia* (6.0/census), but were more likely to prefer the former.

Selective feeding of leaves among tree species is documented in tree-leaf eating arboreal mammals such as primates, rodents, and marsupials, and the effects of leaf chemical compositions on their selectivity have been examined [4]. Among the leaf chemicals, the secondary metabolites such as tannins (mostly represented as total phenolics) function partly as toxins and partly as inhibitors of protein and carbohydrate digestion [2,9,23,24,25,26]. Mammals tend to avoid leaves highly containing secondary metabolites by their bitter or unpalatable taste [23,27]. In contrast, clear preference for soluble sugar or sweetness have been documented because sweetness is a reliable marker of energy content [28]. Water contents are likely to indicate leaf softness. Mammals possibly prefer to eat leaves containing more water if they avoid harder leaves [29]. In our study, a strong preference for *Q. acutissima* and moderate for *P. serratifolia* seems to be related to leaves containing more glucose and water than leaves of *Q. acutissima* (Figure 4). Avoidance of leaves with a high phenolic content was not supported (Figure 4). In the season when *Q. acutissima* is in exploitation, leaves of *P. serratifolia* containing more glucose and less phenolics are likely to be preferred than leaves of *Q. sessilifolia* (Figure 4). Thus, our previous prediction that leaf glucose concentration is an important factor affecting which species of tree leaves the flying squirrels eat [13] was confirmed by the comparisons among the three tree species.

### 4.2. Favorite Parts of a Leaf and Microscale Chemical Distributions

The flying squirrels exhibit partial consumption of the single leaf [4,12,13]. They drop individual leaves after consuming only the apical, central, or basal part. In central consumption, they make a hole at the leaf center, consuming the corner after folding the leaf two times, first longitudinally and second horizontally [4,12]. This is a rare situation where we can try to examine how microscale distributions of chemicals in the leaf affect the feeding choices of herbivorous mammals. This study revealed that frequencies of leaf debris with apical, central, basal, and marginal consumption differed between the three tree species (Figure 3), and within-leaf chemical distributions also differed among these trees (Figure 5 and Figure 6).

We noticed a new feeding mark (marginal type), consuming only the margin of leaves (Figure 1d). Marginal consumption observed only in *P. serratifolia* leaves may be related to the preference for glucose that is contained more at the leaf margin than the center. Sweet parts may be preferred commonly by mammalian herbivores. In contrast, avoidance of eating leaf margins may be explained by spines arranged along the leaf margin [2,30]. Only *Q. acutissima* has leaf marginal spines among the three tree species; therefore, the effect of leaf spines may be negligible in this study.

Central consumption is frequently observed in *P. serratifolia* and *Q. acutissima*. In *Q. acutissima*, the central consumption may be strongly related to the preference for glucose that is contained more at the leaf center and avoidance for phenolics that are contained more at the leaf margin. However, these relationships are not supported in the central consumption of *P. serratifolia* leaves. In both tree species, water is contained more at the leaf center and the squirrels possibly prefer this part to avoid the hard margin. In *Q. acutissima*, basal consumption is also frequent, compared to other tree species. This basal consumption is also related to water contents because leaves of this tree contain more water at the basal part. In *Q. sessilifolia*, leaves are relatively small and homogeneous in chemical distributions. Leaf-eating mammals are apt to feed from the apical part; therefore, apical consumption may be the most common in leaf debris of *Q. sessilifolia*.

Our previous prediction that microscale distributions of phenolics and water in the leaf affect which parts the flying squirrels eat [13] is not always supported. Preference for the central part that contains more water is consistent with prediction. However, despite different tendencies in phenolic contents between the central and marginal parts of the leaf, the central consumption is frequent both in *P. serratifolia* and *Q. acutissima*. This result suggests that other factors affect their partial feeding of the leaf. Tannins are one of the common phenolics produced as a defensive chemical of plants. Mammalian herbivores adopt physiological countermeasures against dietary tannins, such as producing tannin-binding salivary proteins and degrading tannins by the activity of gut microorganisms [25,26], and acclimation to tannins occurs [31,32]. At present, there is no study of such tannin detoxification in the flying squirrels. If they have tolerance and acclimate to tannins, food selectivity based on tannin concentrations may change according to individual acquisition of tolerance and acclimation by experience. Behavioral factors also limit the central consumption of the leaf. This feeding manner is achieved by a two-times leaf folding [4,12] and needs learning [12]. Thus, individual variation, learned or not learned by past experiences, may affect the frequency of central consumption. In the future, we must pay more attention to the effects of phenolics on herbivores’ selective feeding of leaves with insights of physiological adaptation to harmful tannins and behavioral plasticity from experiences.

## 5. Conclusions

The chemical components of tree leaves differ between tree species, conspecific individuals, leaves of the same individual, and the parts of a single leaf. Therefore, food selection by herbivores is expected to occur in these hierarchical levels. The Japanese giant flying squirrels are tree-leaf eating herbivores. They drop leaf debris after partial consumption of individual leaves. Leaf debris is distinguished into four types: apically, centrally, basally, and marginally consumed leaves. This situation is a rare case where we can compare the fed and unfed parts of a single leaf based on within-leaf chemical distributions. In this study, phenolic, glucose, and water contents were measured and compared between apical and basal parts of the leaf and between central and marginal parts. The three tree species, evergreen *Phonitia serratifolia*, deciduous *Quercus acutissima*, and evergreen *Q. sessilifolia*, were used as the main food of the flying squirrels in the study site. In comparisons of preference and chemical contents among the three tree species, leaf glucose concentration was an important factor determining which species of tree leaves the flying squirrels eat. In comparisons between preferred parts and chemical contents in the single leaf, preference for the central part was related to higher water contents at the leaf center than the margin. However, despite different tendencies in phenolic contents between the central and marginal parts of the leaf, the central consumption was frequent both in *P. serratifolia* and *Q. acutissima*. This result suggests that factors other than leaf chemicals affect the frequencies of four types of partial feeding. The physiological tolerance and acclimation to phenolics and the ability of leaf central consumption that needs learning to fold the leaf twice may affect what parts of the leaf they eat.

## Data Availability

The datasets generated during and/or analyzed during the current study are available from the corresponding author upon reasonable request.
